# Microstructural Features and Microhardness of the Ti-6Al-4V Alloy Synthesized by Additive Plasma Wire Deposition Welding

**DOI:** 10.3390/ma16030941

**Published:** 2023-01-19

**Authors:** Irina P. Semenova, Yuri D. Shchitsyn, Dmitriy N. Trushnikov, Alfiz I. Gareev, Alexander V. Polyakov, Mikhail V. Pesin

**Affiliations:** 1 Laboratory of Multifunctional Materials, “Higher Engineering School of Aerospace Materials” Center, Ufa University of Science and Technology, 32 Zaki Validi St., Ufa 450076, Russia; 2 Department of Mechanical Engineering Innovative Technologies, Perm National Research Polytechnic University, 29 Komsomolsky pr., Perm 614990, Russia

**Keywords:** titanium alloy, additive manufacturing, plasma wire deposition welding, microstructure, mechanical properties

## Abstract

Wire arc additive manufacturing (AM) is able to replace the traditional manufacturing processes of Ti alloys. At the same time, the common drawback of Ti workpieces produced by AM via wire deposition welding is the formation of a coarse-grained dendritic structure, its strong anisotropy and, consequently, lower strength as compared to a monolithic alloy. In this work, a new method is proposed for the enhancement of the strength properties of the Ti-6Al-4V alloy synthesized by AM via wire deposition welding, which involves the use of a wire with an initial ultrafine-grained (UFG) structure. The UFG wire is characterized by a large number of defects of the crystalline lattice and grain boundaries, which will enable increasing the number of “crystallization centers” of the α-phase, leading to its refinement. The macro- and microstructure, phase composition and microhardness of the Ti-6Al-4V alloy samples were investigated. The microhardness of the alloy produced by layer-by-layer deposition welding using a UFG wire was shown to be on average 20% higher than that of the samples produced by a deposition welding using a conventional wire. The nature of this phenomenon is discussed, as well as the prospects of increasing the mechanical characteristics of Ti alloys produced by additive manufacturing.

## 1. Introduction

Titanium alloys are widely applied in various industries, in particular, in medicine and aviation, owing to their high specific strength, corrosion resistance, low elastic modulus [1,2,3,4]. In present-day conditions, the manufacture of titanium semi-products and products using the conventional processes of forging, die forging and casting requires considerable expenses related to production planning. The use of additive manufacturing enables the acceleration of the solution of process tasks and the reduction of expenses related to the release of final products [5,6,7]. On an industrial scale, AM has limited use and is applied mainly during the repair of complex metallic products by means of local restoration using a metallic powder or a wire [6].

Most of the studies in the area of additive manufacturing using a wire deal with steels of different series [8]. The application of additive manufacturing with layer-by-layer deposition welding using a wire made of Ti and Al alloys is conditioned by the need to produce lightweight and rugged segmented structures, e.g., an aircraft fuselage. However, the experience and development activities regarding wire-based manufacturing of components made of Ti alloys are relatively limited to date. At the same time, there are a number of publications demonstrating that wire arc additive manufacturing may replace the traditional manufacturing process of Ti alloys. However, during this process, a coarse β-grain forms, which may accumulate in several layers of the deposited material, leading to a strong texture and anisotropy, and correspondingly, to low values of strength and hardness as compared to a conventional monolithic material [8,9,10,11,12,13].

It is known that, the strength properties of Ti alloys depend on the morphology of the α-phase and the sizes of the β-grains that are varied by the material’s temperature and heating rate, as well as by the cooling rate [2]. Apparently, the lower the heat input is during the deposition welding, the higher is the rate of heating and cooling, the smaller is the β-grain size, the larger is the volume fraction of the “harder” α-phase, and the higher is the hardness of the synthesized alloy [13]. The search for possible solutions to this problem is presented in [14,15,16,17,18,19]. In particular, the authors in [14] studied the effect of the cooling rate of the deposited material on the microstructure and tensile properties. It was shown that under cooling, fine α  +  α′ grains were observed and tensile properties and hardness were also improved. The authors in [15] investigated the effect of heat input on the microstructure and mechanical properties of the Ti-6Al-4V alloy samples produced by wire arc additive manufacturing (WAAM). A low heat input (5 × 10^5^ J/m) transformed the columnar grains to equiaxed grains owing to the accelerated solidification rates, thereby improving the alloy’s strength characteristics. The paper [16] investigated the influence of two different deposition strategies, oscillation and parallel pass, on the mechanical properties and high-cycle fatigue of a wire + arc additive manufactured Ti-6Al-4V alloy. In the oscillation build, the plasma torch and the wire feeder continuously oscillated across the wall thickness direction. At 10^7^ cycles, a fatigue strength of 600 MPa was achieved for the oscillation build vertical samples and the parallel pass build. The papers [17,18] investigated the efficacy of combining a rolling step sequentially with layer deposition in the WAAM of large-scale parts for macro-β grain refinement. The paper [19] reported on the results of the study on the additive formation of products from a high-temperature Ti-Al-V alloy using a hybrid technology of multi-layer surfacing, CMT (Cold Metal Transfer), with layer-by-layer strain hardening (CMT Advanced), which enabled a reduction of the β-grain size and, correspondingly, an increase of the strength of the deposited material.

In the present work, a new method is proposed for the enhancement of the strength properties of the Ti-6Al-4V alloy synthesized by additive manufacturing via wire deposition welding which involves the use of a wire with an initial ultrafine-grained (UFG) structure. It is known [20,21] that grain size reduction in metals to values in a range of 0.1–1.0 μm by severe plastic deformation (SPD) leads to a considerable strengthening of the material due to an increase in the length of intergranular boundaries and an increase in dislocation density, i.e., the involvement of the grain boundary and dislocation mechanisms of strengthening. The idea of the proposed approach lies in the fact that liquid melt is genetically associated with the initial crystalline state of a material, in particular, by the nature of the interatomic bonds and the formed “short-range order” [22,23]. It can be assumed that during a relatively fast heating, in the liquid melt in the region of above-liquidus temperatures, the non-equilibrium groups of atoms inheriting the features of phase structure are preserved, which further precipitate during cooling [23]. Thus, the use of a welding wire with a UFG structure, which is characterized by a large number of defects of the crystalline lattice and grain boundaries, will increase the “crystallization centers” of the α-phase, and thereby, can result in a significant refinement of the structure of the grown workpiece and, consequently, an increase in a set of its mechanical characteristics.

The aim of this work is to study the possibility of increasing the strength of grown workpieces through layer-by-layer plasma deposition welding using a wire made of the Ti-6Al-4V alloy with a UFG structure. A UFG structure was produced in the Ti-6Al-4V wire by ECAP–Conform and subsequent drawing [24]. The plasma deposition welding was conducted using a standard commercial wire and a UFG wire and a substrate of the same alloy. The macro- and microstructure of the deposited material was studied, and its microhardness was tested.

## 2. Material and Methods

### 2.1. Substrate and Wire Material

To produce a welding wire and a substrate, commercial hot-rolled rods of the (α + β) Ti-6Al-4V titanium alloy (Grade 5 according to ASTM B348) with a diameter of 12 mm were used. The alloy’s chemical composition is presented in Table 1.

The temperature of the alloy’s polymorphic β→α + β transformation was 989 °C according to the manufacturer’s certificate.

### 2.2. Producing Welding Wire with an Ultrafine-Grained Structure

**Figure 1:**
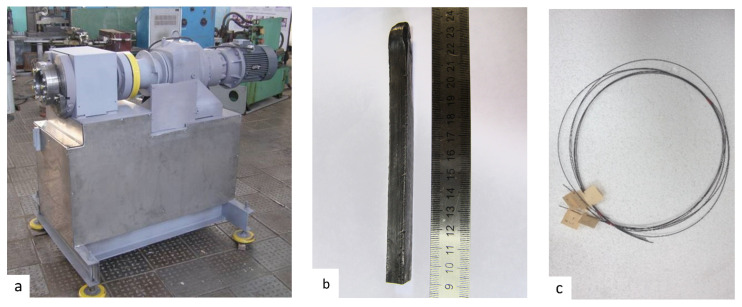
General view (**a**) of the ECAP-C-600 facility; (**b**) view of the Ti-6Al-4V alloy rods produced using the ECAP-C-600 facility; (**c**) view of the wire with a diameter of 1.5 mm.

The deformation of the rods was carried out using an ECAP-C-600 facility (Figure 1a).

The ECAP-C processing regimes are presented in Table 2.

The temperature of drawing from 12 to 1.5 mm was 450 °C, which is the optimum temperature for a satisfactory work of the lubricant; the drawing rate was 0.9 mm s^−1^. The deformation degree in one pass was 10 to 23% depending on the diameter of the workpiece subjected to processing. Generally, the lowest deformation degree is selected for smaller diameters in order to exclude sample fracture during drawing. The total deformation degree after ECAP-C and drawing reached 70%. The wire was subjected to annealing at 300 °C for 2 h. As a result of the drawing process, wires with a diameter of 1.5 mm and a length of over 1 m were produced; their view is shown in Figure 1c.

### 2.3. Deposition Welding Equipment, Regimes and Principles

The deposition welding was carried out using a plasma torch for layer-by-layer welding, developed at Perm National Research Polytechnic University (Perm, Russia). The description of the used equipment is given elsewhere [25].

**Figure 2:**
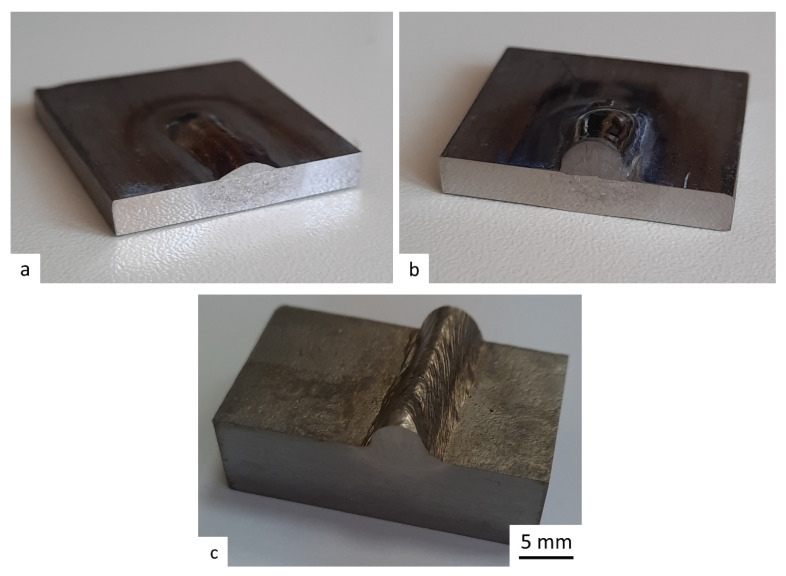
View of a sample after 1 layer (**a**) and 3 layers (**b**) of deposition welding using a UFG wire and (**c**), a layer of CG (conventional wire).

The deposition welding was carried out through plasma straight-polarity deposition welding with a non-consumable electrode. The regime parameters were as follows: plasma-forming nozzle diameter *d*_pn_ = 3.4 mm, arc current *I* = 120 A, plasma-forming gas flow rate *Q*_p_ = 2.0 l/min, shielding gas flow rate *Q*_s_ = 7 l/min, deposition rate *v*_d_ = 25 m/h, wire diameter *d*_w_ = 1.6 mm, wire feed rate *v*_w_ = 4 m/min. The workpiece samples after 1 and 3 layers of deposition welding using a UFG wire are presented in Figure 2.

### 2.4. Study of the Macro- and Microstructure

Samples for metallographic studies were subjected to mechanical grinding, polishing with the use of silicon suspensions and etching with the use of solutions of the following composition: 10(HNO_3_) + 30(HF) + 60(water/glycerol).

For a qualitative and quantitative macroanalysis of the structure, an Olympus GX51 optical microscope with photomicrographic system DP71 (Olimpus, Japan) was used. To study the macro- and microstructure, a JSM-6390 scanning electron microscope (JEOL, Japan) was employed. The mean grain size *d*_mean_ was determined by calculating grain intersections with a confidence level of 0.95 in accordance with the state standard GOST 21073.3-75. The phase fractions were determined from the contrast of the BSE SEM images. The calculation was carried out by pixels.

To study the microstructure of the thin layers (foils) of the Ti-6Al-4V alloy, a JEOL JEM-2100 transmission electron microscope (JEOL, Japan) was used. The work was carried out at an accelerating voltage of 200 kV. The thin foils of the alloy for electron-microscopic studies were prepared by mechanical thinning with an abrasive material. Further, by means of twin-jet electropolishing using a Tenupol-5 unit (Struers, Denmark), a zone with a thickness decreasing towards the center was formed in the disc sample. In this process, an electrolyte was used with the following composition: 6(HClO_4_) + 60(CH_3_OH) + 34(C_4_H_9_OH). The polishing was conducted at a temperature in a range of −25 °C to −30 °C. The voltage during electropolishing was 25 V.

### 2.5. Microhardness

**Figure 3:**
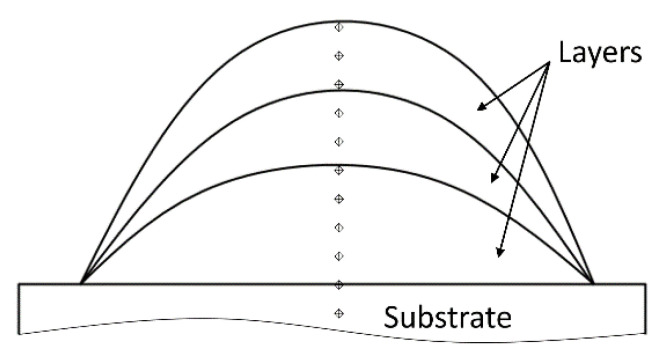
Scheme for measuring microhardness in the cross section of the studied samples (see Figure 2).

The microhardness of the polished samples of the Ti-6Al-4V alloy was tested by the Vickers method using a Micromet-5101 facility (Buehler, Lake Bluff, IL, USA), with a load of 100 g and exposure time of 10 s. The processing of the impressions from a diamond pyramid indenter after load removal and microhardness calculations were carried out using the Omnimet Imaging System software. The principle of microhardness measurement in the section of the samples under study is presented in Figure 3.

The microhardness was measured in the cross cut of the samples through the deposited layers and the substrate. Schematically, the measurements were made along the vertical line in the peak part of the section. Measurements started from the top layer at a distance of 150 microns from the surface and then, with an interval of 500 microns going to the substrate. For each measurement point shown in the diagram, there were several measurements in the nearest area, but not less than five.

## 3. Results and Discussion

### 3.1. Initial Microstructure of the Welding Wire

**Figure 4:**
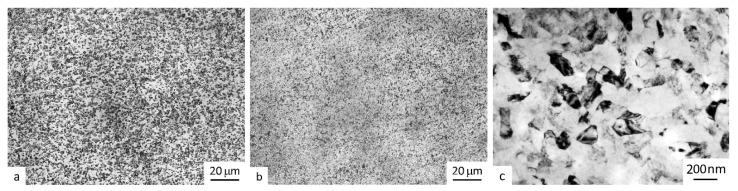
Optical microscopy images of the microstructure of the wire (**a**) from a conventional coarse-grained Ti-6Al-4V alloy and (**b**) from an ultrafine-grained Ti-6Al-4V alloy; (**c**) TEM image of an ultrafine-grained Ti-6Al-4V alloy. Transverse section.

Figure 4a shows the microstructure of the wire produced by a commercial method of isothermal drawing (normally, at temperatures not lower than 700 °C) [26,27]. The wire’s microstructure is characterized by equiaxed grains of the primary α-phase with a size of 3 ± 1 μm.

The structural studies of the wire produced with the use of SPD reveal that drawing produced a fine-dispersed structure with the α-phase size of 0.2 ± 0.05 μm, i.e., it was a typical ultrafine-grained structure [21] (Figure 4b,c).

The microhardness of the UFG welding wire prior to the deposition welding was higher than that of the commercial wire and amounted to HV 466 ± 35 (in comparison to HV 360 ± 35 for the commercial wire).

### 3.2. Macrostructure of the Deposited Material of the Samples

**Figure 5:**
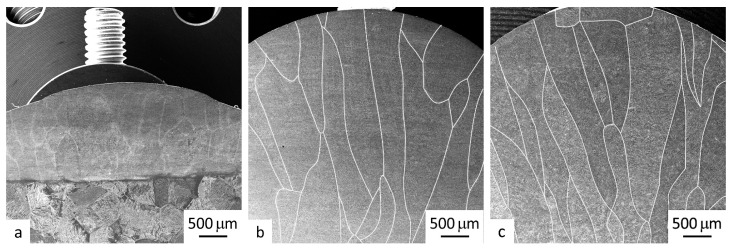
Typical macrostructure in the section of the samples produced by layer-by-layer deposition welding using a wire with a UFG structure in 1 layer (**a**), 3 layers (**b**) and a CG (standard) wire in 3 layers (**c**).

Figure 5 shows the macrostructure of the sample section after 1 and 3 layers of deposition welding using wires. Judging by the macrostructural images, the width of the prior β-phase macrograins in the case of 1 layer of deposition welding is practically identical in both samples (UFG and CG wires) and lies in a range of 300 to 400 μm (Figure 5).

The subsequent layers of deposition welding have resulted in the formation of columnar grains, the nuclei of which are the grains of the first layer. Grain growth occurs along the heat flow gradient perpendicularly to the interface of the substrate, it being a heat sink. According to Figure 5b, in the sample’s macrostructure, after 3 layers of deposition welding using a wire with a UFG structure, there can be seen columnar macrograins about 400 μm wide. There are no distinct interfaces found between the first, second and third layers due to the epitaxial grain growth through the weld layers, following the largest temperature gradient [26]. The internal structure of the grains is very weakly expressed in the macrostructure. In Figure 5c, in the case of deposition welding using a commercial CG wire, a similar picture was observed after 3 layers of deposition welding. Unlike the previous sample (Figure 5b), the internal structure of the macrograins has a more pronounced contrast, which may be related to the formation of large structural elements. A more detailed microstructural study of the deposited material was performed by scanning electron microscopy (see Section 3.3).

It should be noted that such a macrostructure of the Ti-6Al-4V alloy samples produced by additive manufacturing via wire deposition welding is typical and was described in detail elsewhere, e.g., in [13], where deposition welding with a high power laser was used.

### 3.3. Microstructure of the Synthesized Workpieces and Microhardness

**Figure 6:**
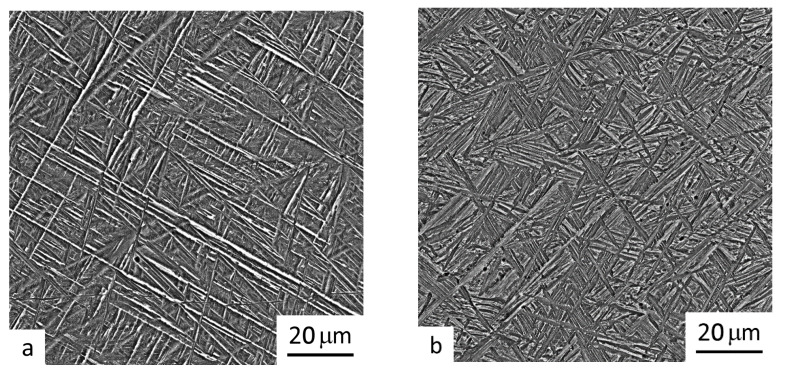
SEM images of the intragranular microstructure of the samples produced by layer-by-layer deposition welding in 3 layers using a wire with a conventional structure (**a**) and UFG structure (**b**).

**Figure 7:**
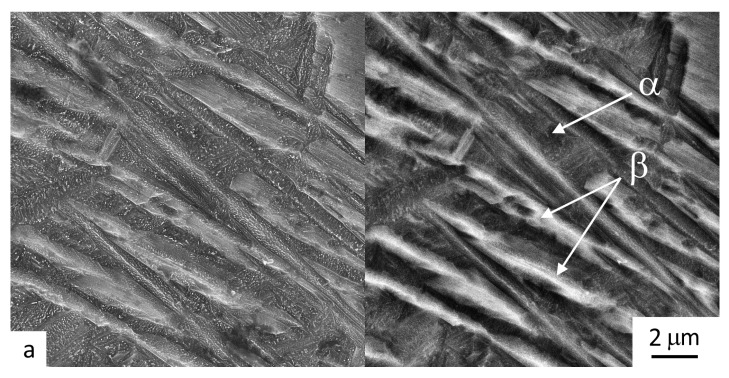

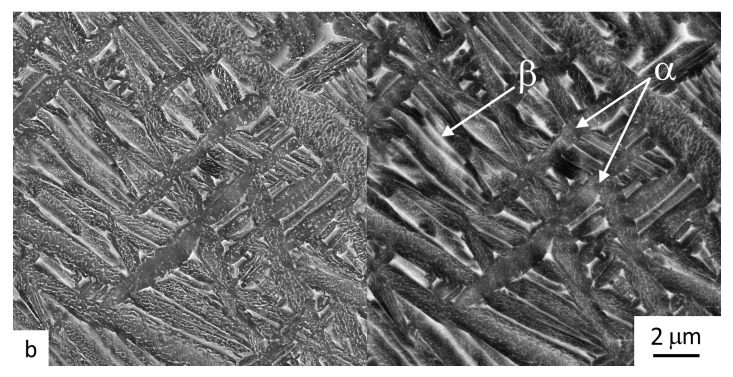
Microstructure in the section of the samples produced by layer-by-layer deposition welding using (**a**) a standard wire in 3 layers and (**b**) a wire with a UFG structure in 3 layers; (left) secondary electrons (SE) mode; (right) mode of backscattered electrons (BSE) in phase contrast.

The intragranular microstructure of the material deposited in 3 layers using CG and UFG wires is shown in Figure 6 and Figure 7, respectively.

It can be seen in Figure 6 and Figure 7 that the interior region of the macrograins in both samples consists of the α-phase lamellae (gray shades in micrographs) in the β-matrix (white contrast in micrographs). The lamellar structure is a consequence of the martensitic transformation of the β-phase during the rapid cooling of the solidified material layer from a temperature above the β-transus temperature. The heating of the solidified layer during the subsequent deposition of the second and third material layers leads to martensite decomposition into the α- and β-phases, with the α-phase retaining its lamellar morphology [13]. The microstructure is characterized by a “basket weave” of the lamellae. This is related to the fact that regions with the β-phase that formed during the cooling of the first layer experience diffusion decomposition when subjected to heating at temperatures in the two-phase region of the phase diagram during a subsequent layer of deposition welding. This leads to the formation of secondary shorter lamellae. Such a microstructure is typical for an alloy synthesized by wire deposition welding via different methods, including that using a high-power laser [13].

That being said, some differences were found between the microstructures of the samples produced by deposition welding using wires with initial CG and UFG structures. Firstly, when a conventional wire was applied, in the sample’s microstructure there were observed, alongside with a “basket weave” structure, regions with large colonies of the α-phase lamellae (compare Figure 6a,b). Secondly, differences were observed between the sizes of the secondary lamellae in the samples under study. In the sample produced by deposition welding using a UFG wire, the length of the lamellae was in the range 8–10 μm (Figure 6b and Figure 7b). In the case of the deposition welding using a CG wire, the lamellae lengths were noticeably larger and reached the size of a grain (Figure 6a and Figure 7a). The more dispersed structure in the case of a UFG wire is an unusual factor since the formation of the structure of the deposited metal passes through the liquid phase. UFG materials are characterized by a higher density of non-equilibrium boundaries, subboundaries, dislocations and other defects of the crystalline structure [20]. It can be assumed that during a relatively fast heating, in the liquid melt in the region of above-liquidus temperatures, the non-equilibrium groups of atoms inheriting the features of phase structure are preserved, which further precipitate during cooling [23]. Apparently, such clusters and non-equilibrium atomic groups are centers for the nucleation of the α-phase during a subsequent cooling of a deposited layer. In this case, an increase in the number of such centers leads to the formation of thinner and shorter α-phase lamellae.

It should also be noted that the volume fraction of the matrix β-phase in the sample produced using a UFG wire is noticeably smaller than that in the sample after deposition welding using a CG wire (7 and 15%, respectively). This is confirmed by the BSE images of the microstructure of the samples taken by a scanning electron microscope (see Figure 7). A probable reason for such a difference could be the fact that in the SPD-processed wire, the β-phase fraction is normally lower (below 5%) than in the equilibrium composition due to its diffusion decomposition under severe plastic deformation [27].

**Figure 8:**
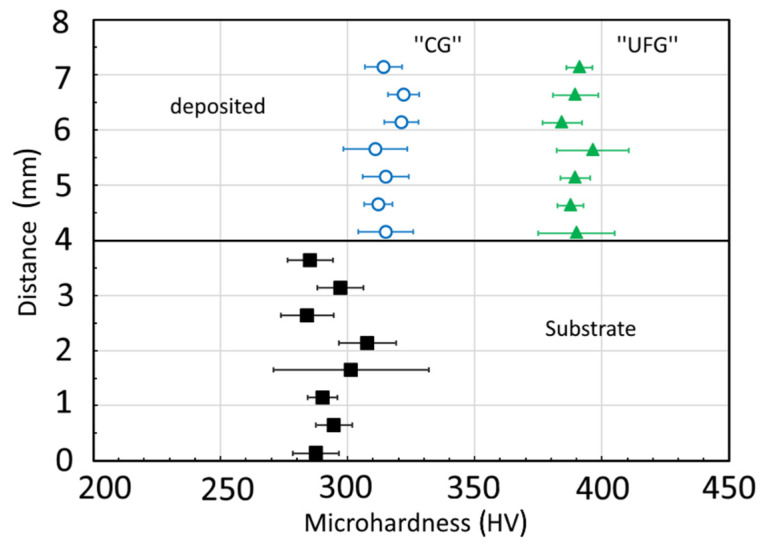
Microhardness in the section of the Ti-6Al-4V alloy samples produced using standard (CG) and UFG wires.

The results of the microhardness tests of the samples under study are presented in Figure 8.

The microhardness value is measured in the direction from the substrate to the upper boundary of the third layer (Figure 8). The microhardness value in the top portion of the samples is 20% higher than that near the interface of the substrate (Figure 8). This fact is characteristic of a metal synthesized by AM wire deposition welding [8]. Comparing the microhardness of the samples under study, it can be seen that the microhardness of the material produced using a UFG wire is higher than the microhardness of the sample produced using a standard wire (400 ± 15 and 320 ± 20 HV, respectively), which can be attributed to the formation of a more dispersed lamellar structure in the former case.

Table 3 presents the mechanical properties of the Ti-6Al-4V alloy obtained in this work in comparison with the results previously obtained, in particular, by additive manufacturing via layer-by-layer deposition using a high power diode laser [12,13], wire arc additive manufacturing with an oscillating regime of wire feed [16], the hybrid technology of multi-later surfacing-CMT (Cold Metal Transfer) with layer-by-layer strain hardening (CMT Advanced) [19].

As is known, the lowest strength is typical for the cast Ti-6Al-4V alloy (834 MPa), and the deformed semi-products have higher strength properties (880–1080 MPa) that depend on the deformation temperature and cooling rate [1]. It can be seen from Table 3 that layer-by-layer wire deposition welding enables achieving strength comparable with the deformed state of the material, as it was demonstrated in [12,16]. The approach proposed in the present work, which involves the use of an initial high-strength wire with a UFG structure, enables the production of the maximum microhardness up to 400 HV in the deposited material. If we use the known ratio between the hardness HV and ultimate tensile strength (HV = 2.8–3.0 σ_UTS_), it is practically satisfied for the alloy in [16]. In this case, it can be assumed that the ultimate tensile strength of the deposited material obtained in the present work with the use of a UFG wire may reach a value of 1120 MPa. That being said, this assumption requires experimental validation for workpieces consisting of more layers (over 3). Here, the previously studied regimes and approaches that have a beneficial effect on the morphology of the formed structure will be applied. In particular, recent studies demonstrate the prospects of plasma deposition welding with a consumable electrode (Plasma-MIG). It is a hybrid process that combines consumable-electrode arc welding and plasma welding which enables one to regulate the heat input into the products in a wide range, increases productivity to 10 kg/h and above, and provides high quality [28,29]. In addition, the introduction of deformation during the layer-by-layer synthesis of a workpiece leads to grain size reduction, a decrease in the anisotropy of the macro- and microstructure and, consequently, an increase in the mechanical characteristics of a workpiece [25].

## 4. Conclusions

Based on the results of the present study of the Ti-6Al-4V alloy workpieces produced by AM via deposition welding using wires with conventional coarse-grained and ultrafine-grained structures, the following has been established:

1. The macrostructure of the samples after 3 layers of deposition welding in both cases consisted of columnar prior β-grains with a thickness of 400 μm due to their epitaxial growth along the heat flow gradient, perpendicularly to the interface of the substrate.

2. The formed microstructure of the molten metal grown with the use of CG and UFG wires was characterized by a lamellar morphology of the α-phase in prior β-grains as a result of a martensitic transformation during the cooling of the crystallized metal.

3. The microhardness values of the metal deposited with the use of a wire with a UFG structure were found to be higher than the microhardness of the sample produced with the use of a CG wire (HV and 400 ± 15 and 320 ± 20, respectively). The achieved effect of increased microhardness is associated with the more dispersed morphology of the lamellae of the α-phase in the metal deposited using a UFG wire and apparently conditioned by the preservation in the melt of non-equilibrium groups of atoms inheriting the features of phase structure that serve as centers for the nucleation of the α-phase during the subsequent cooling of the deposited layer.

## Figures and Tables

**Table 1:** Content of alloying elements in the Ti-6Al-4V alloy (wt.%).

Al	V	Fe	O	C	N	H
6.02	4.00	0.19	0.154	0.019	0.008	0.0033

**Table 2:** ECAP-C regimes used for producing the Ti-6Al-4V alloy rods (Figure 1b) with a UFG structure.

Parameters	Values
Sample section prior to deformation, mm	Ø12
Sample section after deformation, mm	11.5 × 11.5 ± 0.3
Workpiece temperature, °C	500 ± 5
Die-set temperature, °C	600 ± 5
Pressing route	Bc
Number of processing cycles, pcs.	4

**Table 3:** Mechanical properties of the Ti-6Al-4V alloy obtained by additive manufacturing via layer-by-layer deposition.

	Work [12]	Work [13]	Work [16]	Work [19]	This Work	Cast [1]	Rolled Rod [1]
UTS, MPa	942 ÷ 987	-	981 ± 13	-	-	834	880–1080
HV	330 ± 10	327 ± 10	350 ± 10	229 ± 25	400 ± 15	-	-
